# Association of dietary selenium intake and all-cause mortality of Parkinson’s disease and its interaction with blood cadmium level: a retrospective cohort study

**DOI:** 10.1186/s12877-024-05000-6

**Published:** 2024-05-10

**Authors:** Xinyu Tu, Na Wu, Ying Wan, Jing Gan, Zhenguo Liu, Lu Song

**Affiliations:** 1 School of Medicine, Shanghai Jiao Tong University, No.227 Chongqing Rd (S), Shanghai, China; 2 Department of neurology, Xinhua Hospital, Shanghai Jiao tong University School of Medicine, No.1665 Kongjiang Rd, Shanghai, China

**Keywords:** Selenium, Cadmium, Parkinson’s disease, NHANES, Dose–response, Interaction

## Abstract

**Background:**

Parkinson’s disease (PD) is a slowly progressive neurodegenerating disease that may eventually lead to disabling condition and pose a threat to the health of aging populations. This study aimed to explore the association of two potential risk factors, selenium and cadmium, with the prognosis of Parkinson’s disease as well as their interaction effect.

**Methods:**

Data were obtained from the National Health and Nutrition Examination Survey (NHANES) 2005–2006 to 2015–2016 and National Death Index (NDI). Participants were classified as Parkinson’s patients by self-reported anti-Parkinson medications usage. Cox regression models and restricted cubic spline models were applied to evaluate the association between PD mortality and selenium intake level as well as blood cadmium level. Subgroup analysis was also conducted to explore the interaction between them.

**Results:**

A total of 184 individuals were included. In full adjusted cox regression model (adjusted for age, gender, race, hypertension, pesticide exposure, smoking status and caffeine intake), compared with participants with low selenium intake, those with normal selenium intake level were significantly associated with less risk of death (95%CI: 0.18–0.76, *P* = 0.005) while no significant association was found between low selenium intake group and high selenium group (95%CI: 0.16–1.20, *P* = 0.112). Restricted cubic spline model indicated a nonlinear relationship between selenium intake and PD mortality (P for nonlinearity = 0.050). The association between PD mortality and blood cadmium level was not significant (95%CI: 0.19–5.57, *P* = 0.112). However, the interaction term of selenium intake and blood cadmium showed significance in the cox model (P for interaction = 0.048). Subgroup analysis showed that the significant protective effect of selenium intake existed in populations with high blood cadmium but not in populations with low blood cadmium.

**Conclusion:**

Moderate increase of selenium intake had a protective effect on PD mortality especially in high blood cadmium populations.

## Background

Parkinson’s disease (PD) is a neurodegenerative disease with heterogeneous clinical manifestations. However, resting tremor, rigidity, bradykinesia and postural instability are the classical motor features. Sporadic PD usually evolves gradually over many decades before the emergence of movement symptoms [1]. PD is histologically characterized by the loss of dopaminergic neurons in the nigrostriatal pathway and the formation of Lewy Bodies, the main component of which is α-synuclein [2]. The precise pathogenetic mechanisms underlying these histological changes are not fully comprehended. However, previous studies indicated that oxidative stress, mitochondrial dysfunction and abnormal protein handling play a central role in PD pathogenesis, potentially triggered by the interaction between susceptibility genes and environmental factors [3].

Antioxidants have been noticed for their potential benefits in PD patients, as oxidative stress is thought to play a crucial role in the development of PD. Selenium (Se) is an indispensable trace mineral essential for maintaining immune function, production of active thyroid hormone, adjusting mood states and facilitating fertility [4]. There are a number of indications that selenium is important to the brain. Low or descending selenium intake in some regions, such as Europe, arouse researchers’ concern to selenium [4]. Selenium acts as a cofactor in the form of selenocysteine for numerous proteins and enzymes. Some of these proteins and enzymes possess antioxidant activity. Various selenoproteins have been identified, including glutathione peroxidases (GPxs), selenoproteins N, S, P, W and R, thioredoxins and others [5]. Glutathione peroxidases (GPxs), one of the most abundant antioxidant enzymes in brain, reduce hydrogen peroxide and phospholipid peroxides by catalyzing glutathione (GSH), thereby reducing oxidative stress [5]. Selenium has also been shown to scavenge oxidative stress through metal-binding mechanisms [6]. In animal models of Parkinson’s disease, selenium administration increased levels of antioxidant enzyme and GSH, reduced dopamine loss, maintains cellular DNA integrity and improved motor function recovery [7–9].

Cadmium (Cd) is known to induce oxidative stress and ranked as the seventh most harmful heavy metal among environmental pollutants [10]. Exposure to cadmium occurs mainly in occupations such as mining and electroplating. However, daily exposure to cadmium, which is found in food and tobacco smoke, should not be overlooked [11, 12]. Cadmium accumulation also causes cellular oxidative stress and organ damage in multiple systems [13], including the nervous system [14]. Cadmium may induce oxidative stress by inhibiting antioxidant enzymes, displacing redox-active metals, and inhibiting the electron transport chain [15]. While there are limited laboratory studies on cadmium exposure and PD in animals, there is evidence that such exposure affects the distribution of α-synuclein aggregates, reduces the clearance rate of α-synuclein aggregates, and increases its toxicity [16, 17].

Based on the above laboratory evidence, selenium likely has a protective effect against Parkinson’s disease due to its antioxidant properties. Conversely, cadmium may be a risk factor for Parkinson’s disease because of its capacity to induce oxidative stress. However, results from epidemiological studies have been inconsistent regarding the effects of selenium [18–21] and cadmium [22–24] exposure on PD. Furthermore, it has not been reported whether selenium and cadmium interact in the progression of Parkinson’s disease. Therefore, this cross-sectional study, utilizing the National Health and Nutrition Examination Survey (NHANES) database, aimed to establish whether selenium intake and blood cadmium level are risk factors for PD mortality, as well as to investigate the potential interaction between selenium intake and blood cadmium level on PD mortality.

## Methods

### Study population

Participants were recruited from the 2005–2006 to 2015–2016 NHANES, which is a cross-sectional survey for all individual civilians in US. NHANES is one of the projects of National Center for Health Statistics (NCHS), which is a department of the Centers for Disease Control and Prevention of America (CDC).Data provided by NHANES are available for researchers worldwide. In every two-year cycle, NHANES selected participants by a complex multi-stage probabilistic sampling design. Participants were firstly required to finish questionnaires at home, then undergo physical and laboratory examinations in mobile examination center (MEC). Informed consent was well provided by every participant. Further information is available on NHANES website [25].

**Fig. 1:**
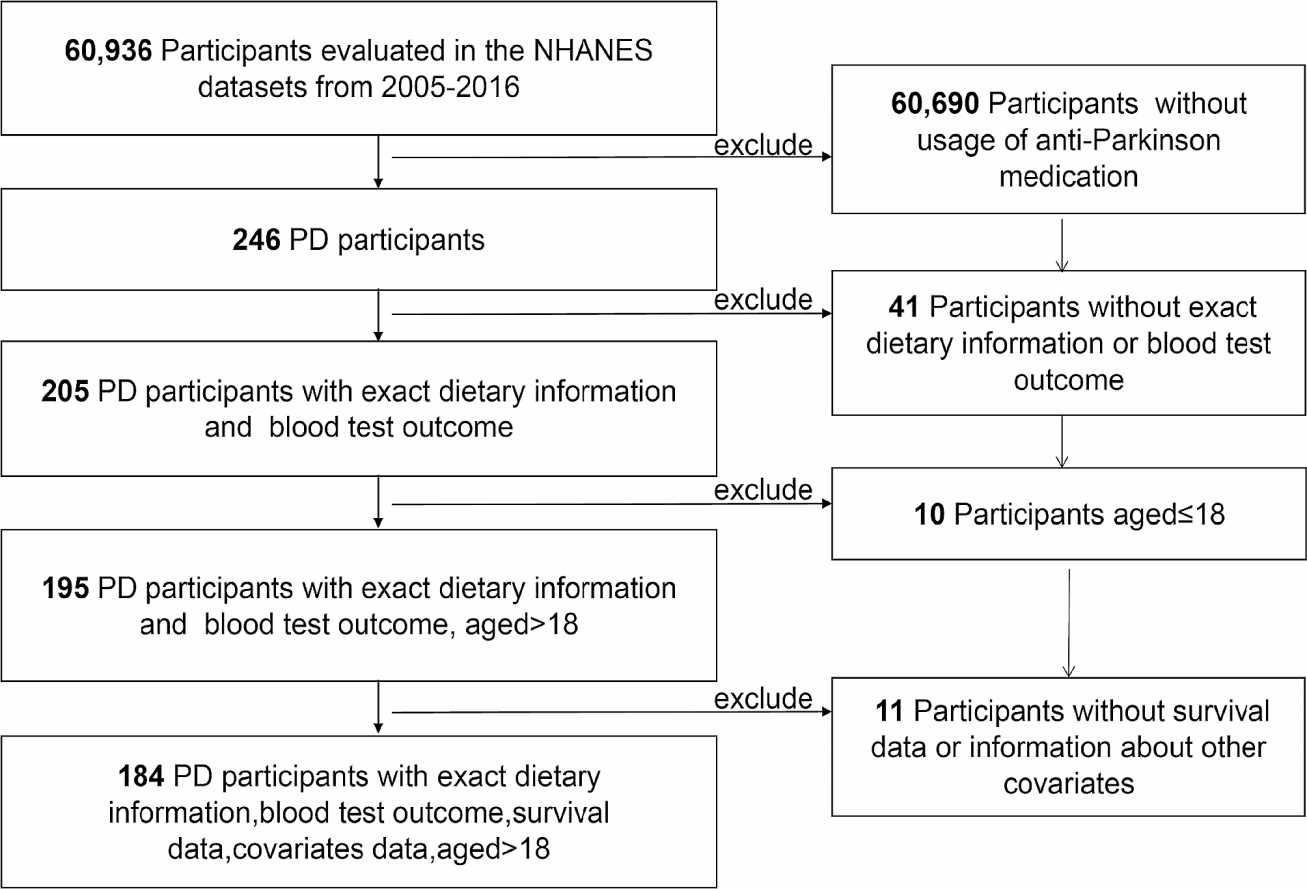
Flowchart of study population selection

In NHANES 2005–2006 to 2015–2016, there were totally 60,936 participants recruited in NHANES project. We firstly excluded participants who were not with Parkinson’s disease (*n* = 60,690). Then participants without exact dietary information or blood test outcome (*n* = 41) were excluded. Participants who aged 18 years or younger was not qualified for this study (*n* = 10). Finally, participants without survival data or information of other covariates were also excluded (*n* = 11). In this study, 184 participants were eventually selected (Fig. 1).

### Definition of Parkinson’s patient

PD cases were determined through self-reported anti-Parkinson medications usage in NHANES database (RXQ_RX module). In NHANES survey, participants were asked whether they have taken prescription medicine in the past 30 days (vitamins or minerals were not included). Those who answered “yes” were then asked for more details, including generic drug name, number of days taken medicine et al. In our study, PD patients were defined as those who reported using following anti-Parkinsonian medications in the past month: Benztropine, Carbidopa, Levodopa, Methyldopa, Ropinirole, Entacapone, Tolcapone, Amantadine, Cabergoline, Orphenadrine and Pramipexole. This method was widely used in previous studies that applied NHANES database to study Parkinson’s disease [26–33].

### Survival data

For NHANES 2005–2006 to 2015–2016, survival status at the end of 2019 was attained through linkage to the National Death Index (NDI), a database of death certificate records. NDI provides mortality files linked to NHANES, which contain information about the survival status and time from NHANES interview to death or end of follow-up. Data are available on [34]. In this study, the overall survival time was calculated by the number of days taken PD medications reported at NHANES interview plus days from NHANES interview to death or end of follow-up.

### Dietary and supplemental selenium intakes

In NHANES database, dietary data were collected with two 24-hour recall interviews. Data were then used to calculate the total intakes of energy, nutrients, and other trace elements (DR1TOT module, DR2TOT module) in the past 24 h. The first 24-hour recall interview was conducted in mobile examination center while the second one was carried out through telephone couple of days later. Participants were asked to provide information about the types and amounts of foods and beverages (including all types of water) consumed during the 24-hour period prior to the interview (midnight to midnight). Each food (or beverage) has a corresponding food code provided by U.S. Department of Agriculture (USDA). USDA also provides the content of various nutrients and trace elements in the food represented by USDA food code. Information was recorded in FNDDS Nutrient Values file, which is available on FNDDS Documentation and Databases of USDA website. NHANES utilized data from USDA to calculate the dietary intake of nutrients and trace elements, including selenium. In our study, dietary intake of selenium per day was calculated by averaging the data of both two interviews. For some participants who did not participant the second interview, data of the first interview alone was used to represent dietary selenium intake per day. NHANES also calculated nutrients and other trace elements intake from dietary supplements during the past 30 days using similar method (data recorded in DSQTOT module during 2007–2016, in DSQ2 module during 2001–2006). In this study, daily supplementary intake of selenium was also taken into account. The total daily selenium intake was calculated by summing the daily dietary intake and daily supplementary intake of selenium.

### Blood cadmium level

Blood samples were collected from participants at the mobile examination center. Samples were then processed, stored, and transported to the Division of Laboratory Sciences, National Center for Environmental Health and Centers for Disease Control and Prevention for analysis. The concentration of blood cadmium was determined by quadrupole inductively coupled plasma mass spectrometry (ICP-MS) technology using ELAN series DRC spectrometers. In this technology, Inductively Coupled Plasma (ICP) is utilized to transform the sample into an ionic state. The high-temperature plasma generated by the ICP is capable of completely evaporating, atomizing, and ionizing the sample, forming positively charged ions. Subsequently, these ions are introduced into a Quadrupole Mass Spectrometer for separation and detection. A Quadrupole Mass Spectrometer is an instrument based on the separation of ions according to their mass-to-charge ratio, which allows specific mass ions to pass through and reach the detector while obstructing or deflecting ions of other masses. Q-ICP-MS combines the efficient ionization capability of ICP with the high selectivity and sensitivity of the Quadrupole Mass Spectrometer, enabling this technology to accurately measure elemental concentrations and isotope ratios in various samples at trace levels. However, this technology has a limit of detection (LODs), and blood metal concentrations below this limit cannot be detected. In some cases, where the result was below the LODs, NHANES database present the result with detection limit divided by the square root of two. The limits of detection were 0.19 µg/L in NHANES 2005–2010, 0.16 µg/L in NHANES 2011–2012 and 0.10 µg/L in NHANES 2013–2016. In this study, blood cadmium levels of 20 participants (10.9%) were below the LODs.

### Covariates

In this study, information about demographic characteristics, lifestyle and healthy characteristics that are possibly associated with PD occurrence reported by previous researches [27, 35] were selected as covariates, including: age (continuous variable); gender (male and female); race (non-Hispanic White, non-Hispanic Black, other Hispanic, other race/multi-racial); educational level (less than 9th grade, 9-11th grade, high school grad/GED or equivalent, some college or AA degree, college graduate or above); pesticide exposure (yes, no); smoking status (non-smokers, former-smokers, active-smokers); diabetes (yes and no); hypertension (yes and no); fat intake g/day (continuous variable); caffeine intake mg/day (continuous variable).

Pesticide exposure was defined as self-reported using any chemical products at home to control fleas, roaches, ants, termites, or other insects in the past 7 days. For smoking status, non-smokers were defined as those who smoked < 100 cigarettes during their lifetimes; active-smokers were defined as those who reported to consume ≥ 100 cigarettes during their lifetime and were still currently smoking; former-smokers were defined as those who reported to consume ≥ 100 cigarettes during their lifetime and have presently quit smoking. Hypertension was categorized as “yes” or “no” according to the self-reported questionnaire or blood pressure measurement conducted in mobile examination center. Participants who reported to have ever been told by a doctor or other health professional that he/she had hypertension or had systolic blood pressure > 140mmHg or diastolic blood pressure > 90 mmHg were regarded as having hypertension. Diabetes status was categorized as “yes” or “no” according to the self-reported questionnaire and blood Hemoglobin A1C (HbA1c) measurement conducted in mobile examination center. Participants who reported to have been diagnosed as diabetes or had blood HbA1c ≥ 6.5% were defined as diabetes status. Caffeine intake and Fat intake were calculated in same methodology as selenium intake.

### Statistical analyses

NHANES database used a complex multi-stage sampling method to sample the American population. Instead of random sampling, NHANES over-sampling sub population with important characteristics (such as smokers). Thus, unlike random sampling, demographic characteristics of the subgroups obtained by over-sampling were different from the overall population. Therefore, the NHANES database assigned specific weights to every participant. The weighted average obtained by weight calculation can better represent the actual characteristics of the whole American population. In this study, all statistical analysis had taken this sampling design into account according to tutorials provided by NHANES. Weight variables, including WTDRD1, SDMVPSU and SDMVSTRA, from the NHANES database were collected to accomplish weighted statistical analysis. WTDRD1 represented the weight of participants who took part in the first 24-hour recall interview. SDMVPSU and SDMVSTRA were masked variance unit pseudo-PSU variable pseudo-stratum variable respectively for variance estimation. Since we merged the 2005–2006 to 2015–2016 survey cycles together, weights for the combined 6 survey cycles were calculated as 1/6*WTDRD1.

Initially, we reported the demographic characteristics of the study population according to different selenium intake level. After inspecting the normality and the homoscedasticity of the data we collected, we chose to demonstrate continuous variables with median and interquartile range [median (IQR)], and compared continuous variables using Wilcoxon rank-sum test for complex survey samples. Categorical variables were shown as the number of cases and its weighted portion [n (%)], and were compared using chi-squared test with Rao & Scott’s second-order correction. After that we conducted univariate cox regression analysis. Variables that had significant effect on PD mortality in univariate cox regression analysis or had been widely recognized as risk (or protective) factor of PD were further included for multivariate cox regression analysis. We then utilized multivariate cox regression model to explore the association between selenium intake / blood cadmium level and PD mortality. Model 1 was not adjusted for any covariables. Model 2 was adjusted for age, gender and race. Model 3 was adjusted for age, gender, race, hypertension, pesticide exposure, smoking status and caffeine intake. Firstly, three cox regression models above were used to explore the association between selenium intake/blood cadmium level and PD mortality as continuous variables. Secondly, same analysis was conducted to explore the association between selenium intake / blood cadmium level and PD mortality as categorical variables. Selenium intake and blood cadmium level were categorized based on quintiles. Sine previous in vitro studies have shown that both excessive high or low selenium exposure can be harmful [28], we defined selenium intake below the 20th quantile as low-level selenium intake (0-68.3 mcg/day), above 80th quantile as high-level selenium intake (> 148.0 mcg/day). Between 68.3 mcg/day and 148.0 mcg/day were defined as normal-level selenium intake. Trend test was performed to exam the linear trend in this relation. High-level blood cadmium was defined as blood cadmium above the 80th quantile (> 0.80 ug/L). Under 0.80 ug/L was defined as normal-level blood cadmium. Thirdly, we further assessed the dose-response relationship between selenium intake / blood cadmium level and PD mortality using restricted cubic spline with 4 knots located at the 5th, 35th, 65th and 95th percentiles of the exposure distribution. We calculated P-values of their nonlinearity. Finally, we conducted subgroup analysis to explore the association of PD mortality and selenium intake at different blood cadmium level. The P-value for interaction were computed applying likelihood ratio tests.

All statistical tests were two-tailed and conducted with R v. 4.2.1 statistical analysis software. Adobe Illustrator v2023 was used for figure preparation. *P* < 0.05 was considered statistically significant.

## Results

### Characteristics of the study population and weighted HRs(95%CI) of covariates

From a total of 60,936 cases in NHANES 2005–2006 to 2015–2016, 184 PD participants met our inclusion criteria and were included in this study (Fig. 1). Among 184 included cases, we recorded 68 deaths before the end of follow-up (December 31, 2019). The median age of the study population was 56 years, and males comprised 36% of all participants. Details of characteristics of the study population according to selenium intake level were demonstrated in Table 1. The median (interquartile range) of selenium intake level was 107.37 (88.66, 134.14) mcg/day in the total population, 54.10 (38.56, 59.75) mcg/day in low selenium intake group, 105.37 (95.24, 119.79) mcg/day in normal selenium intake group and 194.66 (165.42, 211.85) in high selenium intake group. Compared with low selenium intake group, normal selenium intake group tended to have higher daily fat intake (*P* < 0.001), higher prevalence in non-smoker (*P* < 0.004), and lower blood cadmium level (*P* = 0.020). Same phenomenon was also found in high selenium intake group compared with low selenium intake group. However, this difference wasn’t found between normal selenium intake group and high selenium intake group (*P* > 0.05).

**Table 1:** Baseline characteristics of participants according to different selenium intake level, NHANES 2005–2016 (*n* = 184)^a^

Variables	Total, *n* = 184^b^	Selenium intake level			*P*-value^c^
		**Low**, *n* = 37^b^	**Normal**, *n* = 111^b^	**High**, *n* = 36^b^	
**Age (years)**	56 (46, 72)	55 (38, 63)	58 (47, 73)	52 (39, 72)	0.627
**Gender**					0.364
Female	101 (64%)	23 (78%)	64 (63%)	14 (58%)	
Male	83 (36%)	14 (22%)	47 (37%)	22 (42%)	
**Race**					0.159
Non-Hispanic White	130 (85%)	23 (76%)	81 (88%)	26 (84%)	
Non-Hispanic Black	25 (7.3%)	7 (16%)	14 (6.2%)	4 (4.7%)	
Other/multiracial	20 (5.9%)	6 (5.3%)	9 (4.3%)	5 (11%)	
Other Hispanic	9 (1.4%)	1 (2.8%)	7 (1.5%)	1 (0.3%)	
**Education**					0.489
Less Than 9th Grade	23 (6.9%)	7 (16%)	19 (13%)	7 (15%)	
9-11th Grade	33 (14%)	3 (11%)	28 (30%)	7 (32%)	
High School Grad/GED or Equivalent	45 (22%)	10 (36%)	24 (17%)	11 (27%)	
Some College or AA degree	45 (30%)	6 (8.5%)	15 (8.6%)	2 (1.0%)	
College Graduate or above	38 (27%)	11 (28%)	25 (32%)	9 (25%)	
**Diabetes**					0.133
No	145 (85%)	27 (78%)	92 (90%)	26 (76%)	
Yes	39 (15%)	10 (22%)	19 (10%)	10 (24%)	
**Hypertension**					0.234
No	113 (56%)	23 (57%)	71 (61%)	19 (40%)	
Yes	71 (44%)	14 (43%)	40 (39%)	17 (60%)	
**Smoking status**					**0.013**
Non-smokers	98 (59%)	14 (49%)	17 (19%)	6 (13%)	
Former-smokers	49 (19%)	11 (22%)	28 (18%)	10 (18%)	
Active-smokers	37 (22%)	12 (28%)	66 (63%)	20 (69%)	
**Pesticide exposure**					0.665
No	171 (95.1%)	36 (98%)	102 (95%)	33 (95%)	
Yes	13 (4.9%)	1 (2.1%)	9 (5.4%)	3 (5.3%)	
**Fat intake (g/day)**	72.8 (45.5, 91.8)	42.0 (32.3, 47.4)	73.5 (53.5, 87.6)	90.1 (53.8, 102.1)	**< 0.001**
**Caffeine intake (mg/day)**	99.3 (36.1, 221.9)	78.5 (20.0, 252.9)	90.0 (42.5, 243.8)	99.6 (30.9, 168.4)	0.753
**Blood cadmium (ug/L)**	0.32 (0.23, 0.58)	0.84 (0.28, 1.22)	0.33 (0.24, 0.54)	0.26 (0.19, 0.41)	**0.007**

^a^The complex sampling design was accounted for when computing median, interquartile range and proportions. ^b^Median (IQR) for continuous; n (%, weighted) for categorical. ^c^Wilcoxon rank-sum test for continuous variables and chi-squared test with Rao & Scott’s second-order correction for categorical

Then univariable cox regression model was applied to explore the association between covariables and PD mortality. There were statistically significant association between PD mortality and age, gender, hypertension, pesticide exposure. PD participants who were older, male, being exposed to pesticide recently, without hypertension tended to have higher risk of death (Table 2).

**Table 2:** Weighted HRs(95%CI) of cofactors in univariable cox regression analysis, NHANES 2005–2016 (*n* = 184)^a^

Variables	HR (95% CI)^b^	*P*-value^c^
**Age (years)**	1.06 (1.03–1.09)	**< 0.001**
**Gender**		**< 0.001**
Female	Ref	
Male	3.69 (1.76–7.76)	
**Race**		0.053
Non-Hispanic White	Ref	
Non-Hispanic Black	1.93 (0.69–5.40)	
Other/multiracial	0.43 (0.08–2.37)	
Other Hispanic	0.63 (0.15–2.57)	
**Education**		0.084
Less Than 9th Grade	Ref	
9-11th Grade	0.52 (0.16–1.76)	
High School Grad/GED or Equivalent	1.09 (0.38–3.13)	
Some College or AA degree	1.09 (0.38–3.13)	
College Graduate or above	0.44 (0.14–1.35)	
**Diabetes**		0.083
No	Ref	
Yes	1.90 (0.92–3.93)	
**Hypertension**		**0.001**
No	Ref	
Yes	0.33 (0.17, 0.65)	
**Smoking status**		0.500
Non-smokers	Ref	
Former-smokers	1.01 (0.32–3.19)	
Active-smokers	0.68 (0.22–2.18)	
**Pesticide exposure**		**< 0.001**
No	Ref	
Yes	3.46 (1.70–7.03)	
**Fat intake (g/day)**	0.99 (0.98–1.01)	0.467
**Caffeine intake (mg/day)**	1.00 (1.00–1.00)	0.317

^a^The complex sampling design was accounted for when conducting univariable cox regression model. ^b^HR = Hazard Ratio, CI = Confidence Interval. ^c^P-value was calculated based on univariable cox regression model

### Association of selenium intake and blood cadmium with PD mortality

Age, gender, hypertension and pesticide exposure showed significant association with PD mortality in univariable cox regression thus were included in multivariable cox regression as covariables. Race showing a borderline significance was also included. Although the effect of smoking status and caffeine intake on PD showed no significance in univariable cox regression in our study, previous researches had proved their importance on PD [35, 36], thus were also included in multivariable cox regression.

Initially, selenium intake and blood cadmium level were included in cox regression models as continuous variables (Table 2). In all three cox regression models, selenium intake and blood cadmium level showed no significant association with PD mortality (*P* > 0.05). We then included categorized selenium intake and blood cadmium level in cox models (Table 3). Compared with participants who had low-level selenium intake, those who had normal-level selenium intake were significantly associated with less risk of death, both in model 2 (95%CI: 0.21–0.93, *P* = 0.030) and in model 3 (95%CI: 0.18–0.76, *P* = 0.005). However, no significant association was found between low-level selenium intake and high-level selenium in all three models. Consistent with that, the P-values for trend test across the three groups in all three models were > 0.05, indicating that there was no linear trend in this relation. The association between PD mortality and blood cadmium level only showed slight significance in Model 2 (95%CI: 1.01–4.56, *P* = 0.048) but no significance in model1 or model3.

**Table 3:** Weighted HRs (95%CI) of Selenium intake and Blood cadmium level in multivariable cox regression analysis

Variables	Cases/Total	Model1^a^	Model2^b^	Model3^c^
**Selenium intake**				
Continuous				
Per 20mcg/day increase	184/184	1.03 (0.87,1.09)	0.95 (0.84, 1.07)	0.98 (0.87, 1.10)
Categorical				
low-level^d^	37/184	Ref	Ref	Ref
normal-level^d^	111/184	0.66 (0.27–1.60)	0.44 (0.21–0.93)*	0.38 (0.18–0.76)**
high-level^d^	36/184	0.77 (0.30–2.01)	0.50 (0.22–1.14)	0.43 (0.16–1.20)
*P* for linear trend		0.599	0.097	0.112
**Blood cadmium**				
Continuous				
Per 1 ug/L increase	184/184	1.05 (0.77–1.43)	1.33 (0.97–1.83)	0.68 (0.20–2.29)
Categorical				
normal-level^d^	149/184	Ref	Ref	Ref
high-level^d^	35/184	1.24 (0.50–3.08)	2.14 (1.01–4.56)*	1.04 (0.19–5.57)

^a^Model1 univariable cox regression analysis. ^b^Model2 adjusted for age, gender and race. ^c^Model3 adjusted for age, gender, race, hypertension, pesticide exposure, smoking and caffeine intake. ^d^Low-level selenium intake: 0-68.3 mcg/day; normal-level selenium intake: 68.3–148.0 mcg/day; high-level selenium intake: >148.0 mcg/day; normal-level blood cadmium: <0.80 ug/L; high-level blood cadmium: ≥0.80 ug/L. *p < 0.05; **p < 0.01

**Fig. 2:**
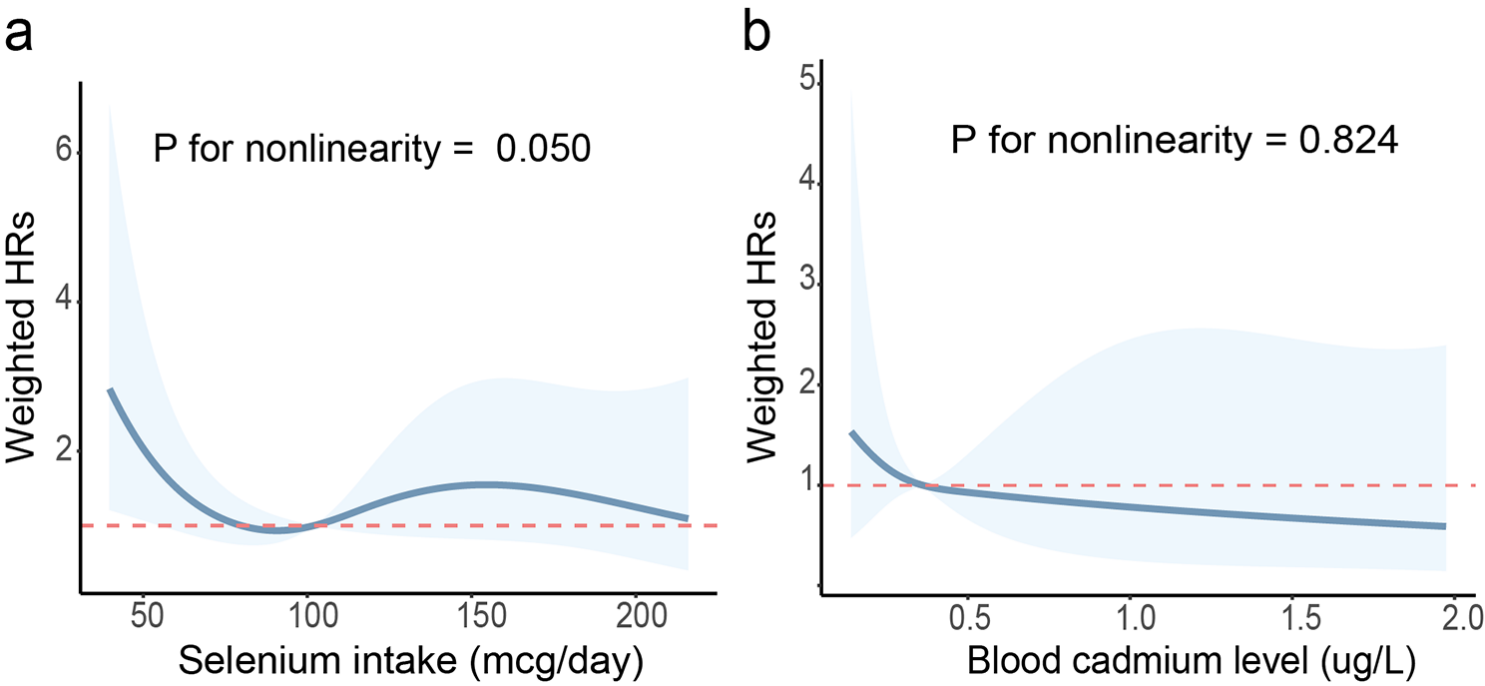
Dose-response relationship between selenium intake (**a**) or blood cadmium level (**b**) and mortality of Parkinson’s patients. The solid blue lines with shaded regions indicate the adjusted HRs and their corresponding 95% confidence intervals respectively. Model adjusted for age and gender, race, hypertension, pesticide exposure, smoking and caffeine intake. (HR = Hazard Ratio)

Additionally, restricted cubic spline model was applied to assess the dose-response relationship between selenium intake / blood cadmium level and their hazard ratios (HRs) (Fig. 2). After adjusted for all cofactors, we found that the effect of selenium intake on PD showed a significance of nonlinearity (P for nonlinearity = 0.050; Fig. 2A). From the RCS curve we observed a steep decrease of HR when daily selenium intake increased from 0 to 95.5 mcg/day, after which curve became relatively plat. The association between blood cadmium level and PD mortality tended to be linear (P for nonlinearity = 0.824; Fig. 2B).

### Interaction between selenium intake and blood cadmium level on PD mortality

**Fig. 3:**
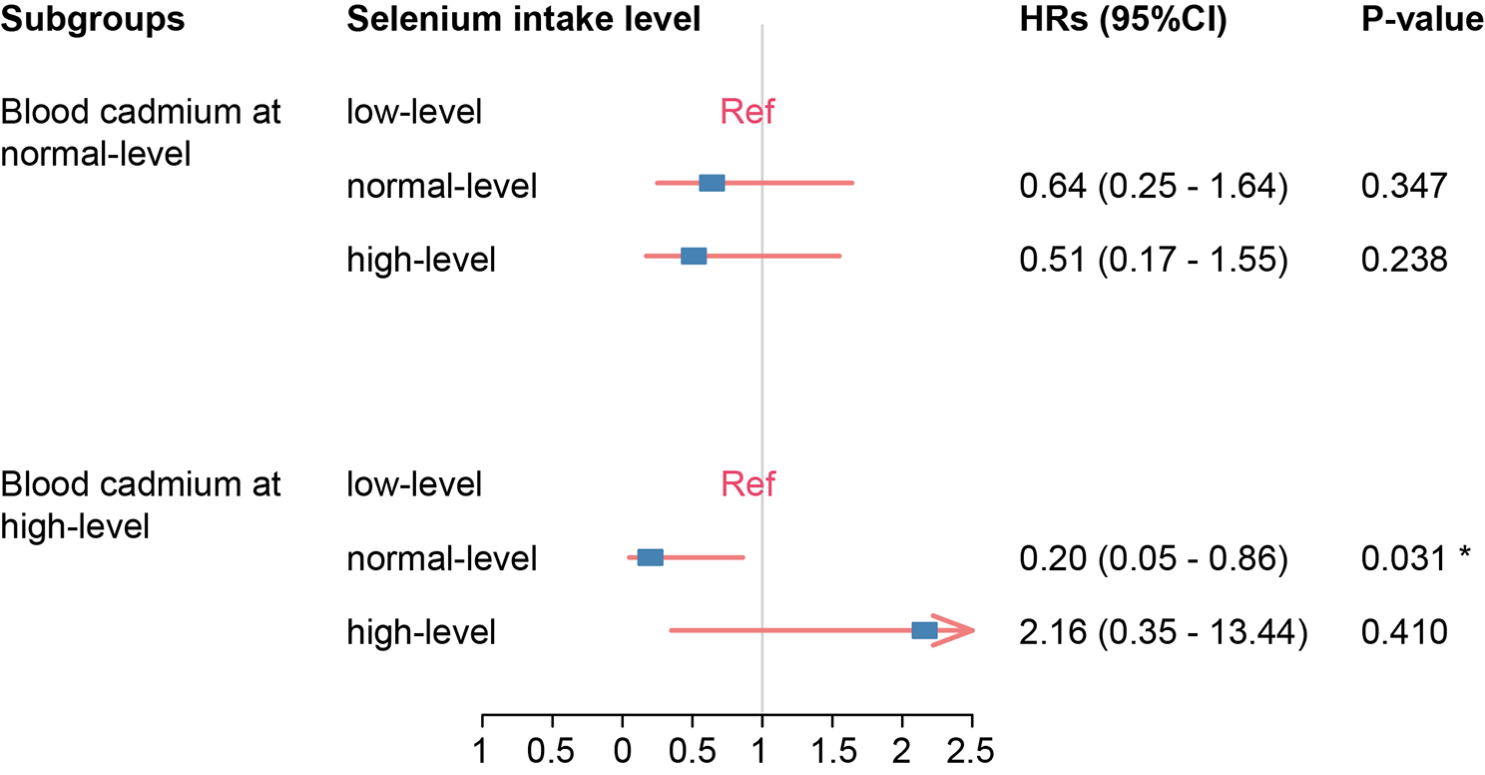
Effect size of selenium intake on PD mortality in blood cadmium-stratified subgroups. HRs(95%CI) were calculated using multivariable cox regression model adjusted for age, gender, race, hypertension, pesticide exposure, smoking and caffeine intake. Low-level selenium intake: 0-68.3 mcg/day; normal-level selenium intake: 68.3–148.0 mcg/day; high-level selenium intake: >148.0 mcg/day; normal-level blood cadmium: <0.80 ug/L; high-level blood cadmium: ≥0.80 ug/L. (HR = Hazard Ratio, CI = Confidence Interval)

Subgroup analysis was conducted in fully adjusted cox regression model. Selenium intake and blood cadmium level were treated as categorical variables. In the group of low blood cadmium level, selenium intake had no significant effect on PD mortality. However, in high-level blood cadmium group, normal-level selenium intake was significantly associated with low death risk (95%CI: 0.05–0.86, *P* = 0.031) compared to low-level Selenium intake. Likelihood ratio test indicated that the interaction between selenium intake and blood cadmium level was significant (P for interaction = 0.048, Fig. 3).

## Discussion

In this cross-sectional, nationally representative sample of adults aged 18 years or older in the US population, we found that normal selenium intake had a positive effect on PD prognosis compared with low selenium intake, but this effect disappeared when compared with high selenium intake. Although there was no association between blood cadmium level and PD mortality, subgroup analysis uncovered that the optimistic impact of selenium consumption on PD prognosis was observed among individuals with high blood cadmium level, but not among those with normal blood cadmium level. These findings propose that moderate enhancement of selenium consumption could improve the prognosis of PD patients, particularly those at risk of high cadmium exposure.

The treatment strategy for PD includes symptomatic therapy and neuroprotective therapy. Symptomatic therapy aims to alleviate symptoms while neuroprotective therapy aims to resist the underlying pathogenesis of PD so as to prevent or delay its progression [37]. Since the free-radical hypothesis has been one of the most influential hypotheses for elucidating the underlying pathogenesis of PD, the administration of antioxidant in PD treatment has been widely paid attention to. Selenium, as a cofactor of several antioxidant enzymes, is an important antioxidant component of human body [5]. Considered the potential effect of selenium against Parkinson’s disease, plenty of case-control studies have explored the relationship between blood selenium level and the incidence of PD [21, 28, 38–43]. Meta-analyses reached a consensus that there was no significant association between blood selenium level and PD [44–47]. However, two of them found that selenium level increased in cerebrospinal fluid in PD patients [44, 46], which was assumed to be an attempt to protect brain against oxidative stress [39, 45]. Above evidence indicated that blood test may not be optimal to reflect selenium exposure in PD. So, we chose daily total dietary selenium intake as our study object. Unlike blood selenium level, there are relatively few studies focusing on dietary selenium intake and PD mortality or morbidity. A case-control study in U.S. reported that high soil selenium concentration helped reduce PD mortality and benefited people with PD in 48 states in America [19]. However, another study focused on inorganic selenium exposure in drinking water in a residential cohort in Italy reported an opposite conclusion [18]. One study conducted in Spain found that the foods frequently consumed by PD patients were rich in selenium, but researchers also realized that these foods also contain abundant copper, which cannot be ruled out as a confounder [20]. Notably, previous studies concerning selenium and PD rarely included covariates in their analysis. Besides, few studies have considered the possible nonlinearity of relationship between selenium and PD. Although several mechanism studies have proved the protective effect of selenium on PD, for example increasing levels of antioxidant enzyme and GSH [48, 49], reducing dopamine loss [7, 8] and maintaining cellular DNA integrity [9], evidence also indicated that different concentrations of selenium have opposite effects on neuron, high dose of selenium is deemed toxic and will in turn promote oxidative stress, inhibitng mTOR activation, causing p62 and ubiquitin accumulation, eventually promoting autophagy [50]. Therefore, we believe that it’s necessary to take the potential nonlinearity of relationship between selenium intake and PD survival into account. In this study, selenium intake was categorized into three groups. After adjusted for all selected cofactors, normal selenium intake level showed a protective effect compared with low selenium intake level, which was not found between high selenium intake and low selenium intake level. The result of trend test did not support liner relationship between categorized selenium intake and PD mortality. Restricted cubic spline model also suggested that there was a significant nonlinear relationship between selenium intake and PD mortality as continuous variable. Our study suggested that for patients with low selenium intake, a moderate increase in selenium intake can reduce the risk of death but excessive high selenium intake can lose this protective effect.

Cadmium is a cumulative environmental poison mainly from occupation, such as mining and electroplating [51]. However, daily exposure to cadmium, such as cadmium contamination in food and tobacco smoke, is also threatening public health [52]. There are also studies indicating that cadmium can accumulate within plants [53] and animals [54] thus impairing food chain. There is evidence that Cd exposure induces oxidative stress [15], blocks mitochondrial electron transport chain [55], induces the release of proinflammatory cytokines [56] in cells. Limited studies have been conducted on Parkinson’s disease and cadmium exposure. However, we found evidence that cadmium affects the distribution of α-synuclein aggregates, reduces the clearance rate of α-synuclein aggregates, and increases its toxicity in PD animal models [16, 17]. This evidence means cadmium may be a risk factor for Parkinson’s disease. However, the result of most of the previous studies did not find a significant association between cadmium exposure and PD morbidity, some even found a negative association between cadmium level and PD morbidity. The findings of a case-control study in U.S. suggested that blood cadmium level was not significantly associated with PD morbidity [41]. Another two case-control studies, which were conducted in India [22] and Italy [57] respectively, found that PD patients have relatively low blood cadmium level compared with the control group. Importantly, all these studies did not include any cofactor in their analysis. Previous studies have also explored the relationship of PD morbidity and cadmium concentration in the environment or in the other part of human body beside blood. Only one study reported a negative association between hair cadmium level and PD morbidity [23], others found no significant difference of cadmium exposure between PD patients and control group [24, 58–60]. One meta-analysis concluded that cadmium level in blood was significantly lower in PD patients, but that in cerebrospinal fluid, serum and urine showed no significant difference in PD patients compared with control group [47]. The mechanism underlying the findings that cadmium level is negatively associated with PD morbidity is still not clear. In Parkinson’s disease, the striatal dopamine level is significantly reduced, resulting in relatively hyperfunction of the acetylcholine system. This imbalance of the neuro transmitter is responsible for the disturbance of the activity of cortico-basal ganglia-thalamic-cortical circuit and the generation of motor symptoms. There is evidence that cadmium can increase the activity of acetylcholinesterase, causing acetylcholine to be hydrolyzed and reducing its concentration [61]. It is possible that this anti-cholinergic effect of cadmium overwhelming its toxicity thus resisting the pathogenesis of PD. We have not found a study that focused on the relationship between blood cadmium level and PD mortality in U.S. population. Our study suggested that the association between blood cadmium level and PD mortality was not significant both as continuous variable and as categorical variable.

In addition, through subgroup analysis we found that the increase of selenium intake did not have a significant protective effect in the normal blood cadmium level subgroup while in the high blood cadmium level subgroup, moderate selenium intake showed a significant protective effect. It is possible that compared with patients with normal blood cadmium level, patients with high blood cadmium level are exposed to more intense oxidative stress in basal ganglia, therefore, the protective effect of selenium is more obvious. Previous studies have not reported the interaction of selenium and cadmium exposure in PD patients or PD animal models, but several studies have reported this interaction effect in plants, neurons and normal animals which were consist with our study. In crops, selenium was proved to be capable of reducing uptake and translocation of cadmium [62]. Se^2−^ [63] and other selenium induced production with abundant sulfur-containing ligands (GSH and phytochelatins) [64] were able to bind with cadmium thereby reducing the upward transport of cadmium in plants. In neurons, selenium pretreatment reversed cadmium-induced decrease of antioxidant enzyme including TrxR1 [65], GSH and GPx [50] and increase of ROS [66]. In rats, pretreatment with Se significantly decreased cadmium-induced elevation of serum brain damage biomarkers, lipid peroxidation, DNA degradation as well as significantly increased the activity and expression of antioxidant biomarkers [67].

This study has several advantages. Firstly, analysis based on NHANES database has several advantages. NHANES database used a complex multi-stage sampling method to sample the American population. It assigned specific weights to every participant for calculating the weighted average of demographic characteristics. Compared with data collected with random sampling, the statistical characteristics of participants in NHANES database can better represent the whole American population. The NHANES database provided a large number of samples, which is important for study of rare diseases like PD. What’s more, NHANES database provided researchers with the amount of each food component consumed by participants in the past 24 h. This allowed us to study the dietary selenium intake and Parkinson’s disease. Besides, NHANES investigated the dietary supplement usage of nutrients. Selenium intake from dietary supplements is an important while often overlooked part of total selenium intake. In this study, up to 49 (26.6%) participants consumed selenium from dietary supplement in the past 30 days. Secondly, we noticed the dual effect of selenium at different concentrations so we included selenium as a categorical variable and analyzed the nonlinear relationship between selenium and PD mortality using RCS curve. We proved that selenium intake at a moderate level rather than high level exerted a protective effect for PD patient. Thirdly, by stratified analyses we found that the protective effect of selenium was significant in patients with high blood cadmium level but not in patients with normal blood cadmium level. To sum up, our study recommends Parkinson’s patients, especially those with high blood cadmium or at high risk of cadmium exposure, to appropriately increase intake of selenium-rich food or dietary supplement. Our study also emphasize that selenium intake should be controlled in moderation and that future researches on selenium and PD should consider the nonlinear relationship between selenium and PD mortality. Our findings also encourage future research to take the cadmium exposure into account when study selenium and PD. However, a few limitations should be noted in our study. First, our study is a historical cohort study. Although exposure took place earlier than outcome, the collection of information is retrospective. Thus, the integrity and accuracy of the data may be impaired due recall bias, and there may still be unknown cofactors although we included covariables as much as possible. Prospective cohort studies or RCT studies are still needed to further test our conclusions. Second, the dietary data in the NHANES were obtained from one or two 24-hour recall interviews, which may not accurately reflect the usual situation. To minimize sampling error, we calculated the average selenium intake of two days for those who accepted the second 24-hour recall interview. Besides, when calculating daily selenium intake from dietary supplements (DS), we calculated the daily average selenium intake from DS over the past 30 days instead of merely average selenium intake from DS reported by two 24-hour dietary recalls. The technology used to detect blood cadmium level has a limit of detection (LODs), and blood metal concentrations below this limit cannot be detected. In this study, blood cadmium levels of 20 participants (10.9%) were below the LODs. This inevitably affected the accuracy of results when blood cadmium was included as a continuous variable. To compensate for that, we also included blood cadmium as a categorical variable. The number of participants in low blood cadmium subgroup exceeded the number of participants below the detection limit, thus LOD did not impact the result of cox regression for categorical cadmium level. Last but not least, NHANES database did not directly provide information on whether participants were diagnosed with Parkinson’s disease, so Parkinson’s patients were screened by drug use records in this study. However, some of these anti-Parkinsonian drugs were also used for treating other disease. For example, Ropinirole and Pramipexole were also used to treat Restless legs syndrome [68]; Orphenadrine was also used to treat muscle spasm [69]. So, it is not sure whether those who were using above anti-Parkinson medication were real PD patients. Fortunately, the incidence of these diseases is not high so we anticipate that it will not have a significant impact on the research findings. Although this methodology has some limitations, it was the best available method to classify Parkinson’s patients for studies based on NHANES database. This method was already widely used in previous studies concerning NHANES database and Parkinson’s disease [26–33].

## Conclusions

In conclusion, our study suggested that total daily selenium intake, including selenium from food and from dietary supplements, has a positive effect on the prognosis of Parkinson’s disease at a moderate level but not at a high level. We applied trend test as well as RCS analysis, the result of which approved the nonlinear relationship between selenium intake and PD mortality. Besides, we found that the protective effect of selenium is particularly obvious in the population that are exposed to higher level of cadmium.

## Data Availability

The datasets used and/or analyzed during the current study are available from the corresponding author on reasonable request.

## Abbreviations

PD: Parkinson’s disease
NHANES: National Health and Nutrition Examination Survey
NCHS: National Center for Health Statistics
CDC: Centers for Disease Control and Prevention
NDI: National Death Index
ICP-MS: Inductively coupled plasma mass spectrometry
LODs: Limit of detection
GPxs: Glutathione peroxidases
Se: Selenium
GSH: Glutathione
Cd: Cadmium
MEC: Mobile examination center
HbA1c: Hemoglobin A1C
IQR: Interquartile range
HR: Hazard ratio
CI: Confidence interval
DS: Dietary supplements
